# Organ transplantation in the modern era

**DOI:** 10.1186/s12871-019-0704-z

**Published:** 2019-03-04

**Authors:** Dmitri Bezinover, Fuat Saner

**Affiliations:** 1 Department of Anesthesiology and Perioperative Medicine, Penn State College of Medicine, The Pennsylvania State University- Milton S. Hershey Medical Center, 500 University Drive, Hershey, PA 17033-0850 USA; 2 Department of General-, Visceral- and Transplantation Surgery, Essen University Medical Center, Hufelandstr 55, 45147 Essen, Germany

## Background

Organ transplantation (OT) is one of most successful advances in modern medicine. For patients with end stage disease, transplantation most often provides their only chance for survival. Even before the first transplant was performed, it was clear that OT could only be successful with a multidisciplinary approach. The history of OT has involved a series of breakthroughs in medicine that has influenced all aspects of health care. As you will see, for nearly a century, the contributions of specialists in anesthesiology and critical were largely underrepresented in the worlds literature.

### Short history of organ transplantation

The earliest descriptions of OT can be found in ancient Greek, Rome, Chinese, and Indian mythology involving bone, skin, teeth, extremity, and heart transplantation [1, 2]. In the sixteenth century, Italian surgeon Gasparo Tagliacozzi used skin transplant for plastic reconstruction. He was the first to describe what we now know is an immunologic reaction when the graft is obtained from a different person. It was only at the end of nineteenth century that OT research began to be both more systematical and better documented. The first animal models (usually dogs) were developed at this time. Early in the twentieth century, French surgeon Alexis Carrel (who later move to the US) developed a new method for vascular anastomoses. Dr. Carrel performed several successful kidney transplants in dogs, developed an approach for vessel reconstruction, and began the practice of cold graft preservation. In 1912 Dr. Alexis Carrel was awarded the Nobel Prize in Physiology or Medicine for his pioneering work [3]. The first human to human transplant was performed in 1933 in the Soviet Union by the Ukrainian surgeon U.U. Voronoy. The blood group mismatched graft was obtained six hours after the donor’s death and although the patient survived two days, the graft never produced urine [3, 4]. Despite significant *surgical* developments, OT was not very successful due to a lack of knowledge in immunology.

The next significant breakthrough in OT came as a result of the work of the British biologist Sir Peter Brian Medawar. His specialty was immunology. During World War II, he worked in the Burn Unit of Glasgow Hospital and investigated problems associated with skin homograft transplantation. For his research on graft rejection and acquired immune tolerance, Dr. Medawar was awarded the Nobel Prize for Physiology or Medicine in 1960 and is considered the father of transplantation.

Between 1951 and 1952, Hume et al., performed nine kidney transplants at the Brigham Hospital in Boston [5]. Despite the use of cortisone for immunosuppression, all grafts were rejected. This problem was successfully overcome by Dr. Thomas Murray who, in identical twins, performed the first successful kidney transplant. The recipient survived 8 years with normal graft function. Dr. Murray received the Nobel Prize for Medicine in 1990. This first success sparked significant enthusiasm in researchers and clinicians in the field of OT. In 1963, after extensive experimental work in animal models, Dr. James Hardy performed the first lung transplant in Jackson, Mississippi. The patient survived for 18 days without any evidence of rejection. Internationally, over the next 10 years, a number of lungs were transplanted. They all had poor outcome related primarily to problems with the healing of surgical anastomoses. The first attempt at liver transplantation occurred in 1963 by Dr. Thomas Starzl, and in 1967, he completed the first successful liver transplant at the University of Colorado. One year later, the first European liver transplant was performed by Dr. Roy Calne in England. Also in 1967, Dr. Christiaan Barnard transplanted the first human heart in South Africa. The recipient was 53 years old and survived for 18 days. Over the next 12 months, more than 100 heart transplants were performed worldwide [6]. Unfortunately, overall survival was poor primarily due to the lack of effective immunosuppression.

In the 1950s, the first attempts at immunosuppression for kidney transplantation involved total irradiation and was met with some degree of success [7, 8]. The use of chemical immunosuppression, initially with 6-mercaptopurine and then with combination of azathioprine and steroids, avoided the problems associated with irradiation and improved outcomes significantly. It was the discovery of cyclosporine in 1976, and its introduction in clinical practice in 1984, that dramatically changed the landscape of OT resulting in an increased one-year survival in both kidney and liver transplant recipients (95 and 75% respectively). Modern immunosuppressive agents (tacrolimus, sirolimis, mycophenolic acid, and everolimus), allow us to currently enjoy superior outcomes and a reduction in adverse immunosuppressive effects.

The next important milestone in the development of OT was the founding of the United Network of Organ Sharing (UNOS) in 1984. This organization manages all transplant activities in the US including the maintenance of a national transplant list for all types of transplantation, data collection, and coordination of educational activities. There are a number of organizations in Europe and Asia with similar responsibilities.

Despite significant contributions in the success of OT, the role of Anesthesia and Critical Care in OT has not always been recognized. As well-known as the names of the first transplant surgeons (Thomas Starzl, Ray Calne, and Russel Strong) are, it is unfortunate that few people are aware that Dr. Antonio Aldrete not only introduced the Postanesthesia Recovery Score and developed the first prototype of the needle for combine spinal/epidural anesthesia but also performed anesthesia for the first liver transplant. Dr. Aldrete was involved in more than 180 liver transplants and described his experience in many publications and lectures. Dr. Thomas Starzl recognized his work as extremely important for the success of transplantation but unfortunately his name is almost forgotten in the history of OT.

In 1992, a group of anesthesiologists and critical care specialists, under leadership of Dr. Yoogoo Kang from the University of Pittsburgh, proposed the creation of a multidisciplinary society to meet the educational needs of medical professionals involved in transplantation and improve the quality of care for transplant recipients. The first two meetings which focused on preoperative care, were held in Pittsburgh in 1984 and 1986. After the success of these first meetings, The International Society for Perioperative Care in Liver Transplantation was created in 1990. It was subsequently re-named The International Liver Transplantation Society (ILTS). At about the same time in Europe, Dr. John Farman founded the Liver Intensive Care Group of Europe (LICAGE). Most recently (2016) The Society for the Advancement of Transplant Anesthesia (SATA) was founded. Today, specialists in Anesthesia and Critical Care increasingly have leadership roles in national and international transplantation societies.

### Specific contributions of anesthesia and critical Care in Organ Transplantation

Advances in anesthesia and critical care, primarily in preoperative evaluation and optimization, intraoperative management, and postoperative care have contributed significantly to the success of OT. The most important contributions have been made in:

- Establishing evaluation and treatment protocols for transplant candidates with comorbidities including CAD, cirrhotic and alcoholic cardiomyopathy, porto-pulmonary hypertension and hepato-pulmonary syndrome, as well as recommendations for the management of hyponatremia
- Introducing the use of perioperative ultrasound and intraoperative TEE monitoring
- The management of coagulopathy, including recommendations on the use of viscoelastic testing and on transfusion component therapy
- Evaluation and management of perioperative hemodynamic instability including post-reperfusion and vasoplegic syndromes
- The management of infections in the immunosuppressed patient

Despite these contributions, transplant anesthesia as a subspecialty is rarely represented at national anesthesia meetings. The situation is similar with the major anesthesia journals. This is changing. ***Anesthesia and Perioperative Care for Solid Organ Transplantation*** is a new section in BMC Anesthesiology and was established to provide the opportunity for anesthesiologists and critical care specialists to present their work in the field of OT. The Section Editors, Drs. Saner and Bezinover have many years of experience in transplantation. They are experts in the field of perioperative care for these very challenging patients and are actively involved the transplant societies ILTS, LICAGE, and The Transplantation Society (TTS).

### Challenges in organ transplantation

Many challenges remain in the field of OT and provide fertile ground for research. The primary challenge in transplantation today for all organ types is the disproportion between organ demand and organ availability. Strategies to overcome this problem include transplantation using extended criteria grafts (ECD), donation after cardiac death (DCD), the use of machine perfusion for graft preservation of inferior quality (or initially discarded) grafts, as well as the use of living donors and split liver grafts. Additional challenges involve perioperative patient care, graft survival, and optimization of immunosuppression protocols. There are several ongoing studies in these areas. There are, however, some specific challenges associated with transplantation of individual organs.

#### Kidney transplantation

There are several areas of research specifically aimed at increasing organ availability and survival to include: optimization of ex-vivo machine graft perfusion and protocols for using extended criteria grafts, preoperative candidate evaluation, graft and recipient matching, pretreatment of recipients (using ischemic preconditioning) and donors (using mild hypothermia) [9]. To help alleviate the shortage of kidneys for transplantation, UNOS has recently introduced a paired donation kidney transplant pilot program. This program helps people who have identified (incompatible) living donors find well-matched donors and receive a transplantation.

#### Liver transplantation

Several strategies have been developed to increase organ availability include living donor liver transplantation (LDLT), split liver transplantation, and utilization of ECD and DCD grafts. The regenerative ability of liver is well known, however in contrast to renal grafts, living donation of hepatic grafts is *significantly* more complicated and puts the donor at greater risk as well. Today, several countries have established LDLT programs with South Korea, Turkey, Japan, and the US being leaders in the field.

Split liver transplantation also offers the possibility to perform two transplantations using one donor. Unfortunately, this option is limited due to the small size of the grafts and can be used only for children and smaller adults.

Other potential options to increase graft availability is the use of ECD, DCD and initially discarded grafts. Utilization of these organs (especially DCD) is not as high as could be due to their lower quality in comparison to donation after brain death organs. There are two major problems associated with transplanting DCD grafts: primary non-function of the transplanted organ [10, 11] and intrahepatic biliary strictures (as a result of ischemic cholangiopathy) [12] due to prolonged warm ischemia time which is unavoidable with DCD donors. Nevertheless, utilization of these grafts is growing. It has been demonstrated that machine perfusion (both normo- and hypothermic) [13–15] during graft preservation can significantly increase their quality, resulting in successful transplants.

Hepatic replacement therapy is also an important area of research. There are a number artificial or bioartificial systems under investigation that may be used as a bridge to transplantation. Hepatocyte transplantation (cell suspension from unused hepatic tissue) also has demonstrated some promise [16]. Currently, these systems have limited efficacy and are topics of ongoing investigations. A *bioengineered* liver is a future concept and is currently under intensive development [17].

#### Pancreas transplantation

The first successful pancreas-kidney transplant was performed in 1966 by Drs. Richard Lillehei and William Kelly at the University of Minnesota. They performed the first singular pancreas transplant in 1968. The pancreas-kidney transplant procedure is very common today due to the high incidence of diabetic nephropathy associated with diabetes mellitus. Isolated islet transplantation is being performed with increasing frequency and is the topic of much ongoing research.

#### Intestinal transplantation

The first attempts at transplanting intestines were performed in 60s. These initial attempts, however, were not successful with the majority patients succumbing to rejection, infections, and surgical complications. Only after introduction of cyclosporine (and later tacrolimus) did intestinal transplantation become possible. The first successful intestine transplant was performed in 1988 by Dr. E. Deltz in Germany. Intestinal transplantation can be performed alone or as a part of multi-organ procedure. Despite significant improvements in survival, rejection and cytomegalovirus infections are still significant problems. The refinement of existing immunosuppressive protocols and the development of new drugs is a priority of research in this field.

#### Heart and lung transplantation

The use of both DCD cardiac and pulmonary grafts was started in the US in 1993. Even though DCD hearts and lungs have been successfully transplanted, [18, 19], the risks associated with using these lower quality grafts is very high. Graft perfusion during preservation (usually normothermic but also hypothermic) of these organs has been demonstrated to be beneficial [18, 20–22].

Other approaches currently in use for cardiac transplantation is acceptance of organs with mild coronary artery disease (CAD) and the use of previously grafted hearts.

Other areas under investigation include preventing and managing chronic rejection, preventing postoperative infection and malignancy, optimization of postoperative outcome, refining surgical techniques, and improving cardiac recovery assessment of donors after hypoxemic events.

#### On the horizon

Xenotransplantation is not new, however, renewed interest in this area is growing and may prove to be a solution for many problems associated with organ storage. In the early 90s, Dr. Thomas Starzl performed 2 baboon to human liver transplants. There remains many unsolved physiological, microbiological, and immunological problems associated with this type of transplantation currently under investigation.

Face, uterus, and extremity transplants have recently demonstrated with some success and will likely be performed with greater frequency in the future. Certainly, the long-term outcome of these patients has to be evaluated.

The new BMC Anesthesiology section, *Anesthesia and Perioperative Care for Solid Organ Transplantation,* was established to provide the opportunity for specialists involved in the care of transplant patients to submit their manuscripts on these topics. We would like to invite Anesthesiologists and Critical Care specialists, as well as all other specialists involved in OT, to submit manuscripts for consideration to this new section of BMC Anesthesiology.

## Abbreviations

CAD: Coronary artery disease
DCD: Donation after cardiac death
ECD: Extended criteria grafts
ILTS: International Liver Transplantation Society
LDLT: Living donor liver transplantation
LICAGE: Liver Intensive Care Group of Europe
OT: Organ transplantation
SATA: Society for the Advancement of Transplant Anesthesia
TTS: The Transplantation Society
UNOS: United Network of Organ Sharing
